# Evolutionary Game Analysis of Government and Residents’ Participation in Waste Separation Based on Cumulative Prospect Theory

**DOI:** 10.3390/ijerph192114589

**Published:** 2022-11-07

**Authors:** Lichi Zhang, Yanyan Jiang, Junmin Wu

**Affiliations:** 1 School of Economics and Management, Jiangsu University of Science and Technology, Zhenjiang 212000, China; 2 School of Electronics and Information, Zhenjiang College, Zhenjiang 212028, China

**Keywords:** waste separation, residents, government, evolutionary game, cumulative prospect theory

## Abstract

Government and residents’ participation in waste separation is a complex non-cooperative game process, and the evolutionary game can explain the behavior of participating subjects well. Considering that the traditional evolutionary game cannot satisfactorily explain the irrational psychology and risk preference factors of the participating issues, this study combines the prospect theory and evolutionary game, uses the prospect value function to supplement and improve the parameters of the evolutionary game payment matrix, and analyzes the evolutionary stabilization strategy. To verify the theoretical results, simulation experiments and impact analysis were conducted, and meaningful results were obtained: There are two stable evolutionary strategies in the system, namely higher participation benefits for residents and lower participation costs and opportunity costs, and reasonable direct benefit distribution coefficients all help to increase the participation rate of waste separation. This study can provide some scientific suggestions for the government to design and build a waste-separation system.

## 1. Introduction

The continuous growth of MSW generation and the related environmental pollution problems have seriously threatened the sustainable development of countries, especially China [1]. Since 2004, the average annual growth rate of MSW generated in China has been 1.13%, surpassing the United States and ranking first in the world [2]. In China, the total amount of MSW has increased from 161 million tons in 2013 to 236 million tons in 2020. MSW has become a major environmental by-product of China’s urban development, and densely populated Beijing and Shanghai have become “cities surrounded by waste” [3]. According to the Chinese Ministry of Ecology and Environment, Shanghai will produce 1076.8 million tons of MSW in 2020, 2.95 million tons per day. Compared to international metropolises, Shanghai’s MSW production is still relatively high. It is also higher than major cities such as Singapore (1.78 million tons/day), New York (1.23 million tons/day), Seoul (1 million tons/day), London (0.99 million tons/day), and Tokyo (0.93 million tons/day) per capita. However, China’s MSW system is still far from perfect compared to other cosmopolitan cities.

Waste separation (garbage classification) is an upgrade to the traditional waste disposal method and a scientific management method to dispose of urban waste efficiently. “In 2017, the General Office of the State Council of China released the Implementation Plan of Domestic Waste Separation, and 46 cities were identified as pilot cities; from 2019, cities across the country began to fully implement waste-separation policy; by the end of 2025, all cities will have established waste-separation systems” [4].

On the other hand, at this stage, waste separation in China usually presents a situation in which the government bears all the costs of waste separation. In contrast, other stakeholders benefit from it without incurring charges; i.e., the problem of “free-riding” emerges [5]. The free-rider problem will prevent the government from choosing more advanced and expensive technologies or lead to the overuse of the services provided. Residents and government are the most critical participants [6]. The evolution of the relationship between the two parties is the focus of this paper, as their preferences for benefits and costs [7] are also taken into account in the behavioral decision-making process.

In addition, school and non-school environmental education and raising environmental awareness of society are essential tasks for the government before implementing changes in waste management—only in this way can the public see and understand immediate and long-term benefits. The process of introducing major changes in national waste management that require public participation must be preceded by public dialogue: a series of promotional campaigns explaining the meaning, need, and benefits of the changes (also environmental benefits, which is why the continuous building of environmental awareness in society is so important).

In practice, the public is not uniform and does not make a homogeneous collective decision about participation in the waste-management system. The factors that determine not only the willingness of the society to participate in household waste separation but also the quality of this separation and the agreement to share in the costs, including financial ones such as payment of a fee for management by local governments of MSW and rental/purchase of appropriate containers, are widely described in the literature based on experience in the implementation of new waste management systems. When analyzing the possibility of changing the MSW-management system, it is necessary to consider the existing solutions to the diagnosed issue worldwide and the lessons learned from their implementation.

Therefore, what kind of impact does the evolutionary relationship between the two parties in the participation in waste separation have on waste separation? What is the best way to develop a reasonable incentive strategy to improve the effectiveness of government policies and the participation rate of residents based on the consideration of benefit and cost preferences, i.e., cumulative prospect theory? Therefore, the purpose of this paper is to analyze the mutual evolution of government and residents’ participation in waste separation based on the cumulative prospect theory and the impact of residents’ participation in waste separation through evolutionary game theory and to explore how to formulate reasonable policies to improve residents’ participation rate to provide a reference for promoting resource recycling and sustainable development.

Scholars’ research on waste separation is divided into four main areas.

(1) Behavior of waste separation: The behavior of waste separation is one of the earliest issues that received academic attention, and domestic and international research on waste-separation behavior has focused on the factors influencing separation behavior [8,9,10,11,12,13], source separation [14,15,16,17], and participant intent [2,12,18,19], and some other scholars have conducted research in the Chinese context.
(2) Waste-separation strategies: With the gradual systematization of behavioral research on waste separation, strategy research on waste separation has also been emphasized by domestic and foreign scholars, with foreign studies focusing on hazardous-waste-separation methods [20,21], waste-separation policy utility [22,23,24], and correctness of waste separation [25,26], among others.
(3) Mechanism of waste separation: The mechanism research of waste separation is an inevitable stage of waste-separation research, and domestic and foreign studies mainly focus on waste-separation management mechanism [27,28,29,30], waste-separation and recycling user mechanism [31,32,33], and construction waste-separation and disposal mechanisms [34,35].
(4) Waste-separation spillover: Studies are more comprehensive regarding waste separation and related studies, but the relationship between waste separation and its external subjects in the supply chain has not been studied much. Domestic and foreign studies have focused more on the relationship between waste separation and the environment [36], economy [37,38,39], rural revitalization [30], etc., and have studied the relationship between waste separation and the external subjects of its supply chain to a lesser extent.

From the review of existing related literature, although a large number of scholars have conducted studies from several levels and perspectives, such as the behavior of waste-separation participants—residents, waste-separation management strategies, and mechanism developers—the government; however, few studies have involved the relationship between residents and the government, specifically, no studies have been conducted from a dynamic change perspective while considering the evolution between the behavioral strategies of residents and the behavioral strategy of the government under the irrational psychology and risk preferences of the participating subjects.

On the other hand, considering that the government and residents are individuals with limited rationality in the actual implementation of waste separation due to information asymmetry, environmental dynamics, and limitations of people’s thinking [40,41,42], the participation of the government and residents in waste separation is a game process of continuous improvement and dynamic evolution.

Given this research status, the primary purpose of this paper is to use a virtual dynamic equilibrium analysis paradigm, i.e., evolutionary game theory, to construct a non-cooperative evolutionary game model between residents and the government’s behavioral strategies, to combine the cumulative prospect theory with a non-cooperative evolutionary game model to analyze the behavioral change process of government and residents and the influencing factors of cumulative prospect theory, to explore how the government can optimize the ratio of the benefits of both sides in waste separation and effectively work together to increase the willingness of residents and government to participate in waste separation, and to provide theoretical references for the government to develop a more rationalized waste-separation policy.

The evolutionary game theory emphasizes evolutionary stable strategies (ESS) of human behavior. It is used to analyze the formation process of human social customs, norms, and institutions and their influencing factors [43]. The evolutionary game is based on Darwin’s biological evolution and Lamarck’s genetic theory rather than the assumption of complete rationality; the evolutionary game model takes the participating group as the object of study, analyzes its dynamic evolutionary process, and then explains why and how the group reaches the equilibrium state. Since the cumulative prospect theory can well explain the irrational psychology and risk preferences of participating subjects when making decisions [7], the use of the prospect value function to supplement and improve the parameters of the evolutionary game payment matrix can provide a scientific analytical paradigm for the same type of research.

## 2. Modeling

### 2.1. Model Assumptions

Without considering the influence of third parties other than the government and residents, the choice of government and residents to participate in waste-separation behavior can be seen as the result of a game between two parties involved in the waste separation, and the game itself can be regarded as a risky decision-making behavior. Therefore, this paper makes the following assumptions:

(1) Behavioral choice: Assume that two subjects—residents and government—are involved in waste separation at the moment. Assume that the participation strategy set of residents is (participation, non-participation), and the participation strategy set of government is {participation, non-participation}. When both residents and government choose to participate, the waste separation is successful. It produces direct transformation to obtain total benefits, which residents and government share, with the sharing ratio of β and 1 − β (0 < β < 1), respectively. At the same time, the implementation of waste separation will lead to the improvement of resource utilization efficiency and the original ecological environment in addition to the provision of public services required by society for waste separation and minimization and resource utilization and the potential benefits gained by the government in investment attraction, talent attraction, environmental management, pharmaceutical R&D, etc., due to the improved environment. When residents decide not to participate, and the government decides not to participate, both parties still do not gain the benefits from waste separation. However, the government still needs to bear the management costs of participating in waste separation.
(2) Participation benefits: If residents choose the “non-participation” strategy, they can obtain the general utility U1. If the government decides on “non-participation”, they can obtain the available utility U2. Suppose both residents and the government choose the “participation” strategy. In that case, they can obtain the total benefit of K, which residents and the government share; the use obtained by residents is βK, and the benefit received by the government is (1 − β)K.
(3) Participation cost: It is assumed that residents’ participation in waste separation requires input cost T (T > 0), including residents’ labor cost for waste separation and the cost of waste-separation tools, etc. The government’s participation in waste separation requires an input management fee of C (C > 0), including labor, device, and management costs necessary for waste separation.
(4) Prospective gains and losses: When residents choose to participate, and the government decides to participate, the government will gain potential profits P (P > 0) due to investment attraction, talent attraction, and urban governance after environmental improvement; at the same time, residents need to bear the opportunity cost of opportunity cost M (M > 0) that may come from participation in waste separation.
(5) Participation probability: the probability of residents taking the “participation” strategy is x, and the possibility of taking the “non-participation” strategy is 1 − x, where 0 ≤ x ≤ 1; the probability of the government taking the “participation” strategy is y, and the possibility of taking the “non-participation” strategy is 1 − y, where 0 ≤ y ≤ 1.

Based on the above five assumptions, the payment matrix of the participation game in waste-separation behavior is constructed.

### 2.2. Constructing the Expected Return Function

According to the payment matrix in Table 1, when residents choose the “participation” strategy, the expected benefits can be found as follows.

(1) $$EA_{1}=y\left(U_{1}+\beta K-M-T\right)+\left(1-y\right)\left(U_{1}-M-T\right)\;=y\beta K+U_{1}-M-T$$

When residents choose the “non-participation” strategy, their expected return is.

(2) $$EA_{2}=yU_{1}+\left(1-y\right)U_{1}=U_{1}$$

The average is expected to return when residents choose a mixed strategy, i.e., the “participation” strategy versus the “non-participation” strategy.

(3) $$EA_{3}=xEA_{1}+\left(1-x\right)EA_{2}$$

Similarly, the expected returns *EA*_1_ and *EA*_2_ when residents choose the “participation” and “non-participation” strategies and the average expected return *EA*_3_ when they choose the mixed strategy are

(4) $$EB_{1}=x\left[U_{2}+\left(1-\beta \right)K-C+P\right]+\left(1-x\right)\left(U_{2}-C\right)\;=x\left[\left(1-\beta \right)K+P\right]+U_{2}-C$$

(5) $$EB_{2}=xU_{2}+\left(1-x\right)U_{2}=U_{2}\;$$

(6) $$EB_{3}=yEB_{1}+\left(1-y\right)EB_{2}\;$$

### 2.3. Solving the Replicated Dynamic Equation

Based on the expected return model above, the replicated dynamic equation for resident (A)’s choice of “participation” strategy can be derived as

(7) $$F\left(x\right)=x\left(EA_{1}-EA_{3}\right)=x\left(1-x\right)\left(EA_{1}-EA_{2}\right)=x\left(1-x\right)\left(y\beta K-M-T\right)$$

The replicated dynamic equation for the government’s (B) choice of “participation” strategy is

(8) $$F\left(y\right)=y\left(EB_{1}-EB_{3}\right)=y\left(1-y\right)\left(EB_{1}-EB_{2}\right)=y\left(1-y\right)\left[x\left(1-\beta \right)K+xP-C\right]$$

From *F*(*x*) = 0 and *F*(*y*) = 0, five local equilibrium points can be found:

$$(0,\;0),\;(0,\;1),\;(1,\;0),\;(1,\;1),\;\left(\frac{C}{\left(1-\beta \right)K+P},\frac{M+T}{\beta K}\right).$$

From the resident government replicated dynamic equation, it can be found that

(9) $$\frac{dF\left(x\right)}{x}=\left(1-2x\right)\left(y\beta K-M-T\right)$$

(10) $$\frac{dF\left(x\right)}{y}=x\left(1-x\right)\beta K$$

(11) $$\frac{dF\left(y\right)}{x}=y\left(1-y\right)\left[\left(1-\beta \right)K+P\right]$$

(12) $$\frac{dF\left(y\right)}{y}=\left(1-2y\right)\left[x\left(1-\beta \right)K+xP-C\right]$$

The Jacobian matrix is obtained by Equations (9)–(12) as

(13) $$J=\left[\begin{array}{cc} \left(1-2x\right)\left(y\beta K-M-T\right) & x\left(1-x\right)\beta K\\ y\left(1-y\right)\left[\left(1-\beta \right)K+P\right] & \left(1-2y\right)\left[x\left(1-\beta \right)K+xP-C\right] \end{array}\right]$$

The values of the determinant of the matrix are

(14) detJ=(1−2x)(1−2y)(yβK−M−T)[x(1−β)+xP−C]+xy(1−x)(1−y)βK[(1−β)K+P]

The traces of the matrix determinant are

(15) trJ=(1−2x)(yβK−M−T)+(1−2y)[x(1−β)K+xP−C]

## 3. Model Discussion

### 3.1. Equilibrium Strategy Stability Analysis

According to the above evolutionary game model, the stability of participation in waste separation can be analyzed in two cases.

First, when βK − M − T > 0, (1 − β)K + P − C < 0 or βK − M − T < 0, (1 − β)K + P − C > 0 or βK − M − T < 0, and (1 − β)K + P − C < 0.

According to the local stability analysis method proposed by [44], four local equilibria are obtained in the system S = {(x, y; 0 ≤ x, y ≤ 1)}, which are (0, 0), (0, 1), (1, 0), and (1, 1). The equilibrium results of the Jacobian matrix are shown in Table 2.

**Figure 1:**
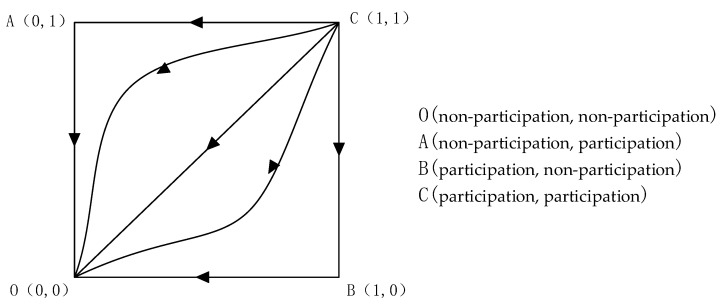
Evolutionary phase diagram when the expected benefit to one of the residents and the government is less than the cost to them.

As can be seen from Table 2, when the expected benefit of one of the residents and the government is less than the cost they pay, points (0, 1), (1, 0), and (1, 1) are unstable points, and points (0, 0) are strategically stable points, and since the expected benefit of one of the residents and the government is less than the cost they pay, the evolutionary game strategy of both sides must be {non-participation, non-participation}, which is also practically compatible. The evolutionary phase diagram is shown in Figure 1.

The second explains the outcome when the benefits received by the residents and the government are more significant than their costs, i.e., βK − M − T > 0 and (1 − β)K + P − C > 0.

It can be obtained that there are five local equilibria in the system S = {(x, y);0 ≤ x, y ≤ 1}, which are (0, 0), (0, 1), (1, 0), (1, 1), and $\left(\frac{C}{\left(1-\beta \right)K+P},\frac{M+T}{\beta K}\right)$. The equilibrium results of the Jacobi matrix are shown in Table 3.

**Figure 2:**
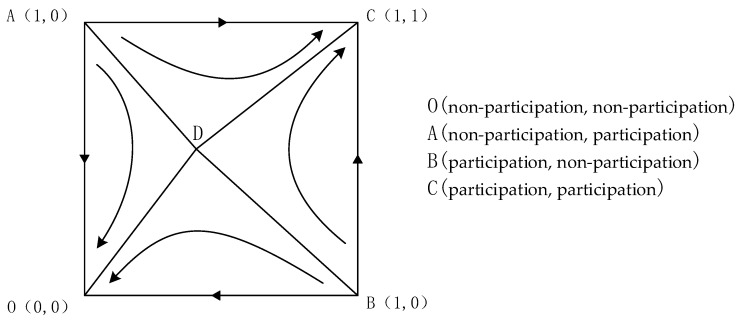
Evolutionary phase diagram when the benefits received by both residents and the government are more significant than the costs paid by them.

As shown in Table 3, points (0, 0) and (1, 1) are stable points when the benefits of both residents and the government are more significant than their costs. They correspond to the two strategies {non-participation, non-participation} and {participation, participation}, respectively. Points (0, 1) and (1, 0) are game instability points and $\left(\frac{C}{\left(1-\beta \right)K+P},\frac{M+T}{\beta K}\right)$ saddle points. The evolutionary phase diagram is shown in Figure 2.

As shown in Figure 2, in the initial state ACDB region, the system converges to the point C (1, 1), the participation willingness of residents and government will evolve to the {participation, participation} strategy; when in the ADBO region, the system will converge to (0, 0), and the participation willingness of residents and government will evolve to the (non-participation, non-participation) strategy.

### 3.2. Evolutionary Game Analysis Based on Cumulative Prospect Theory

#### 3.2.1. Cost–Benefit Function Based on Cumulative Prospect Theory

By analyzing the equilibrium point of the evolving system, it can be seen that the benefits obtained by the residents must be greater than the sum of their time costs T and possible opportunity costs, and the sum of direct and potential benefits received by the government must be greater than its management costs. “When both sides converge at (0, 0), i.e., the Pareto inferior equilibrium point, both residents and the government choose the “non-participation” strategy.” To make both parties converge to the Pareto optimal equilibrium point with maximum probability, the behavior of both parties should assemble to (1, 1).

From assumption (4), when both residents and the government choose the “participation” strategy, there is an opportunity cost in which residents miss other benefits because they spend time participating in waste separation. The probability of missing other benefits is P1. The opportunity cost incurred by residents is M. If missing other benefits does not occur, the opportunity cost incurred by residents is 0. It is known that the opportunity cost of participating in waste separation M is

M = π(P1)V(m) + π(1 − P1)V(0)
and since V(0) = 0, M = π(P1)V(m)

From assumption (4), it can be seen that after participating in waste separation, residents and enterprises can jointly obtain direct benefits K. At the same time, the benefits are not all necessarily immediate after waste separation, requiring the government to recycle waste materials and treat different types of waste differently after separation. Assuming that the direct benefits that can be obtained after participating in waste separation is P2, residents and enterprises with P2 may generate the immediate benefits K. There are (1-P2) that may receive direct benefits of 0. It is known that the direct benefits K are

K = π(P2)V(k) + π(1 − P2)V(0)
and since V(0) = 0, K = π(P2)V(k)

Similarly, from assumption (2), it can be seen that the government can only obtain the potential benefit P from waste separation if both residents and the government participate in waste separation at the same time so that the government can have the possibility of obtaining the potential benefit p with P2 and the possibility of obtaining the potential benefit 0 with (1-P2). It can be seen that the potential benefit P is

P = π(P2)V(p) + π(1 − P2)V(0)
and since V(0) = 0, P = π(P2)V(p)

The above analysis shows that when βK-M-T > 0 and (1-β)K+P-C > 0, the system enters a local steady state and converges to the points (0, 0), (1, 1). These two conditions indicate that the benefits gained by the residents must be greater than the sum of their time and opportunity costs, and the sum of the direct and potential benefits gained by the government must be greater than its administrative costs.

#### 3.2.2. Analysis of Evolutionary Stabilization Strategies Based on Cumulative Prospect Theory

Both residents and the government are characterized by limited rationality. It is known from cumulative prospect theory that when faced with gains, finite, rational people tend to shift to avoid costly losses. Participation in waste separation is a decision with certain cost losses: (1) the possible cost losses from participation in waste separation are more significant than the gains gained; (2) opportunity costs may be incurred from participation in waste separation; and (3) potential benefits are not immediate. Therefore, residents and the government tend to avoid losses, preferring to forego direct benefits rather than incur potential costs. Decisionmakers differ in their sensitivity to gains and losses, leading to a system that may converge to (0, 0) analytically.

(1) Impact of revenue: The premise of the direct benefits of waste separation is that residents and the government participate in waste separation, with residents putting out waste in the first stage and the government disposing of waste in the second stage and realizing the direct benefits through recycling of recyclable materials and special treatment of hazardous waste. Residents usually underestimate the probability P2 of immediate benefits after participating in waste separation, i.e., π(P2) < P2, so that K = π(P2)V(k) < P2V(k), and the actual direct benefits are more significant than the expected immediate benefits.
(2) Impact of cost: The government can reduce the time cost T by raising residents’ awareness of waste separation through preliminary publicity and education and effectively reduce the management cost by optimizing the waste-separation process. However, due to traditional waste-disposal methods, residents are unwilling to participate in waste separation because of the hassle. Residents and the government overestimate the difficulty of completing waste separation, time cost T, and management cost C. The actual costs incurred in time cost (T1 < T) and management cost (C1 < C) are smaller than the expected costs incurred.
(3) Impact of prospective gains and losses: The characteristic of limited rationality of the waste-separation participant overestimates the possibility of opportunity costs occurring and underestimates the chance of potential profits arising. They tend to overestimate the probability of missing other gains P1, i.e., π(P1) > P1, so that M = π(P1)V(m) > P1V(m), and they underestimate the probability of potential gains from participation in waste separation, i.e., π(P2) < P2, so that P = π(P2)V(p) < P2V(p), so the real opportunity cost is smaller than the expected opportunity cost, and the actual potential gain is larger than the expected potential gain.

Among the above influencing factors, (1) indicates that direct benefits from participation in waste separation are underestimated; (2) indicates that time cost T and management cost C are overestimated; and (3) indicates that π(P1) is overestimated, π(P2) is underestimated, real opportunity cost is overestimated, and real potential benefits are underestimated.

βK – M − T = βπ(P2)V(k) − π(P1)V(m) − T < βP2k − P1m − T1
(1 − β)K + P − C = (1 − β)π(P2)V(k) + π(P2)V(p) − C<(1-β)P2k + P2p − C1

That is, the actual participation benefit is underestimated, the waste-separation participation cost is overestimated, the essential participation direct benefit K and potential benefit P are underestimated, and the participation time cost T and management cost C are overestimated so that when βK-M-T > 0 and (1-β)K+P-C > 0, there are two evolutionary strategies for the evolutionary system due to the different sensitivity of both residents and government to benefits and costs.

## 4. Numerical Analysis

This paper investigates the symbiotic evolution of behavioral strategies for resident and government participation in waste separation based on a combination of cumulative prospect theory. Therefore, the model developed in this paper is generic and can be used to discuss the design of system parameters of residents’ and governments’ participation strategies in waste separation in different countries and regions; in addition, the numerical analysis results of one country can provide scientific basis and valuable reference for other countries and regions. Furthermore, the selection of countries with significant influence in the global waste-separation field as simulation objects to visualize and validate the theoretical results can enhance the credibility and persuasiveness of the research results and increase the influence of this study in related fields.

### 4.1. Setting Parameters

In 2020, China Vanke Co., Ltd. in Beijing, China extended waste separation to 135 Vanke communities in 29 cities across China for pilot projects. According to 19 pilot communities of China Vanke Co., Ltd. in Beijing, each community has reduced waste by more than 25%.

In order to explore the influence of various factors on the evolution of the game between the government and residents’ willingness to participate in waste separation under the cumulative prospect theory, this paper uses Matlab2019a software to conduct a simulation analysis, considering the community residents as member A and the government as member B. By varying the values of different parameters, we observed and analyzed the influence of various factors on the government and residents’ willingness to participate in waste separation under the cumulative prospect theory.

Considering that the government and residents pay more attention to waste separation in the process of waste separation, assume that the residents’ willingness to participate is low at the beginning, setting the initial value of x as 0.3, and the government’s willingness is higher than the residents’ because the government’s knowledge of waste separation is higher than the residents’, thus setting the initial value of y as 0.5; since the ultimate goal of both parties involved in waste separation is based on the maximization of revenue, assume that the direct benefit is greater than the transfer cost, and the transfer cost is less than the receiving cost; the initial value of direct benefit K takes the value of 90, the ease of use of waste-separation facilities is 0.5, the distribution degree is 0.6, and the time cost T and the management cost C take the values of 3 and 6, respectively; and the direct benefit distribution coefficient β takes the value of 0.5; the opportunity cost M of residents and the potential benefit P of the government are the uncertain future loss and income, assuming their initial values, setting M = 1 and P = 3. The remaining parameters are kept constant except for the parameter being analyzed in the analysis (Table 4).

### 4.2. Sensitivity Analysis

#### 4.2.1. Direct Income and Its Distribution Factor

**Figure 3:**
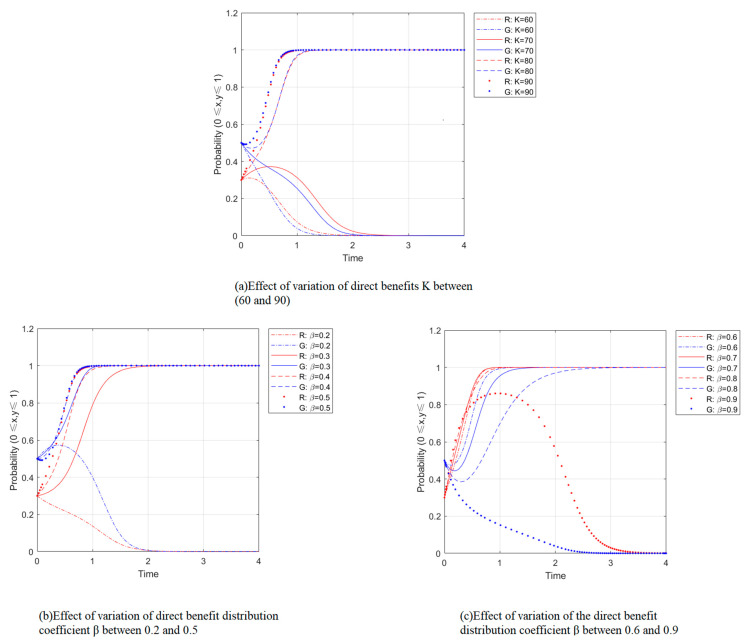
Impact of changes in direct benefits and its distribution coefficient.

Figure 3a shows the simulation of the impact of the change in direct benefits K on the participation strategy of waste separation generated by the simultaneous participation of residents and the government with other parameters constant. As shown in Figure 3a, the critical value of direct benefits is between 70 and 80. x,y converges to 0 when K is less than this critical value, and the final equilibrium point tends to (0, 0). Meanwhile, the increase of K can slow down the convergence to 0; when K is more significant than this critical value, x, y converges to 1, and the final equilibrium point tends to (1, 1). At this moment, the increase of K can speed up the convergence to 1, indicating the direct benefits. This is because the government is responsible for promoting and facilitating the implementation of the central policy and considering the increase in benefits. The simulation results show that the rise in direct benefits makes residents and the government willing to participate in waste separation.

Figure 3b,c shows the simulation of the effect of the change of direct benefit allocation coefficient β on the participation strategy of waste separation by residents and the government with other parameters held constant. From Figure 3c, it can be seen that the critical value of the direct benefit allocation coefficient β is between 0.2–0.3. When β is less than this critical value, x,y converges to 0, and the final equilibrium point tends to (0, 0); when β is more significant than this critical value, x,y converges to 1, and the final equilibrium point tends to (1, 1). At this point, the increase of β makes x join to 1 faster. The convergence of y first accelerates and then slows down the trend, indicating that within a specific range, as β increases, the residents’ willingness to participate in waste separation gradually becomes more assertive. The government’s desire to participate in waste separation is also strengthened and tends to slow down, formed by the government’s function of providing public services. When the benefit distribution coefficient decreases beyond a specific range, the equilibrium point tends to (0, 0), where x converges faster than y, indicating that the benefit distribution coefficient has a more significant impact on residents’ individuality than the publicness of the government. When β is less than this critical value, x,y converges to 1, and the final equilibrium point joins to (1, 1); when β is more significant than this critical value, x,y converges to 0, and the last equilibrium point converges to (0, 0); at this time, the increase of β makes the speed of y slow down. The rate of x does not change significantly. Finally, it converges to 0 after y, which means that when the government gains are too small (less than 0.2 later), the willingness to participate decreases, and the system finally evolves to the strategy of “non-participation” and “non-participation”.

#### 4.2.2. Time Costs and Management Costs

**Figure 4:**
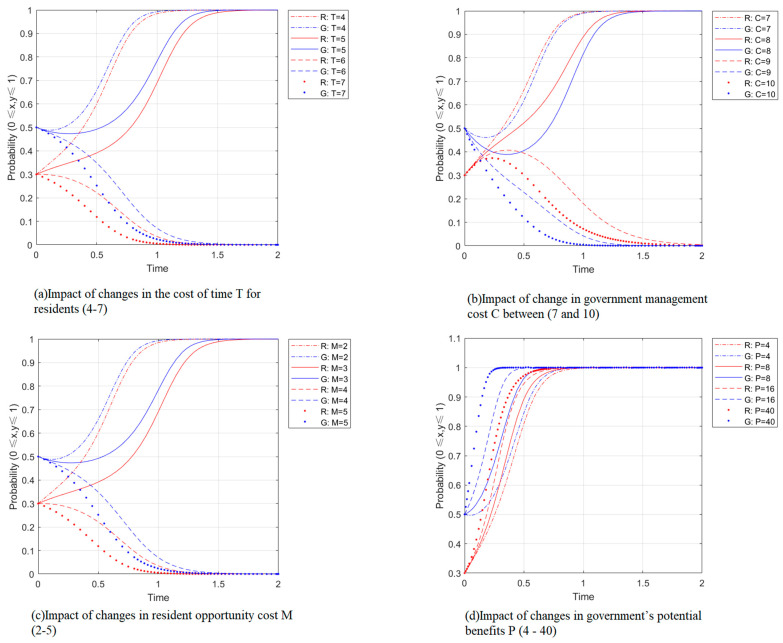
Impact of changes in time costs, management costs, opportunity costs, and potential benefits.

Figure 4a,b shows the effect of the change of residents’ time cost T and government management cost C on the participation strategy of waste separation, with other parameters unchanged. When T is greater than the critical value, x,y converges to 0, and the increase of T accelerates the convergence; when T is less than the critical value, x,y converges to 1, and the decrease of T accelerates the convergence. The smaller the time cost, the more favorable the participation of waste separation; from Figure 4b, it can be seen that the critical value of government management cost C is between 8 and 9. When C is greater than the critical value, x,y converges to 0, then the increase of C makes the convergence slow down; when C is less than the critical value, x,y joins to 1, then the decrease of C makes the convergence faster. The smaller the management cost C, the more favorable the participation in waste separation. It indicates that participation in waste separation is a purpose-oriented behavior, essentially an evolutionary activity that chooses the path of low resistance to proceed. The lower the opposition to participation in waste separation, the more likely it is that participation will occur.

#### 4.2.3. Opportunity Costs and Potential Benefits

Figure 4c,d shows the simulations of changes in the residents’ opportunity cost M and the government’s potential gain P on the waste-separation participation strategy with other parameters held constant. As shown in Figure 4c, the critical value of residents’ opportunity cost is between 2 and 3. When M is more significant than this critical value, x,y converges to 0, and the final equilibrium point tends to (0,0); when M is smaller than this critical value, x,y converges to 1. At this time, the decrease of M makes the convergence speed up, indicating that the larger the residents’ opportunity cost, the lower the willingness to participate. The subtle change in residents’ opportunity cost can cause residents to make completely different decisions; as can be seen from Figure 4d, the evolution of government potential gain P is not significant to the evolutionary results, and even if the government potential gain P increases ten times on the original basis, the residents’ willingness to participate in waste separation does not change significantly.

In contrast, the speed of convergence of the government’s willingness to participate in waste separation to 1 is accelerated, indicating that in increasing potential gain, which happens as the value of possible gain P becomes more significant, the convergence rate of Y is significantly accelerated, indicating that the government’s public interest determines that the government values potentially gain more than the present gain. Cumulative prospect theory suggests that people are sensitive to loss and gain differently, and the pain of loss is much greater than the pleasure of gain. Simulation results show that changes in residents’ opportunity cost M influence residents to make different decisions. At the same time, finite, rational people tend to be less sensitive to future gains than present gains.

## 5. Discussion

This paper combines cumulative prospect theory with the evolutionary game under the premise of finite rationality assumption; analyzes the benefit payment matrix in the evolutionary game model with the benefit function is cumulative prospect theory; incorporates the characteristics of game subjects such as overestimation of probability events, underestimation of probability events, and risk aversion into the evolutionary game analysis; summarizes the behavioral mechanism of residents and government’s participation in waste separation based on the conclusion of this analysis; and draws the following research perspectives.

(1) There are two stable evolutionary strategies in the system. Due to the influence of limited rationality, there are two evolutionary strategies, i.e., {non-participation, non-participation} and {participation, participation}, for waste separation even when the gained benefit for residents is greater than the sum of time cost and opportunity cost. The sum of direct and potential uses for the government is greater than the management cost it pays. Participating agents prefer loss avoidance and forgo immediate benefits rather than bear the potential opportunity costs. In participating in waste separation, participating subjects are more inclined to underestimate the probability of occurrence of direct and potential benefits and overestimate the likelihood of event of opportunity costs, making the system still likely to evolve toward the {non-participation, non-participation} strategy.
(2) Residents are sensitive to participation benefits and participation cost changes. While participation in waste separation benefits residents, it also requires them to bear certain costs or losses. The greater the benefits of participation, the smaller the costs, and the easier it is to participate. The participation cost of residents mainly includes material costs and time costs. Residents need to buy specific waste-separation containers for the project, i.e., material cost; they need to identify and sort the waste before putting it out, i.e., time cost. The government’s participation cost is mainly the human and material cost in the management process, i.e., the management cost. Therefore, by rationalizing and optimizing the design of the waste-separation process, the government can promote residents’ participation in waste separation; reduce the participation cost of both residents and the government, mainly the time cost T and the management cost C; and actively increase the participation rate of waste separation.
(3) The allocation coefficient of direct benefits of participation significantly impacts strategy choice—the distribution coefficient (0 ≤ β ≤ 1) needs to be found between 0 and 1. Simulation results show that too high or too low a distribution coefficient may change the evolutionary strategy from {participation, participation} to {non-participation, non-participation}, thus failing to achieve the goal of both residents and government participation in waste separation. Therefore, the functional departments should design the direct benefit distribution coefficients through legislation or introduce management methods that meet the local conditions to effectively increase the willingness of residents and local governments to participate in waste separation at the same time and realize the purpose of both residents and governments to participate in waste separation at the same time.
(4) The opportunity cost of residents has a significant role in promoting strategy choice. In participation in waste separation, the opportunity cost for residents and the potential gain for the government are both uncertain future losses and gains. The research results show that residents are more sensitive to the performance of opportunity costs. In contrast, the potential gain is not significant to the strategic choice of both participating subjects, which is consistent with the characteristics of cumulative prospect theory. Small-probability events are given larger weights, while medium- and high-probability events are given smaller ones. In the case of participation in waste separation, the resident’s involvement in the waste-separation process causing them to miss other benefits is a small-probability event. The simulation results show that some minor changes make the residents sensitive due to being given larger weights by limited, rational people.

## 6. Conclusions

This paper intends to give the following recommendations from the perspective of exploring how the government can increase the willingness of residents and the government to participate in waste separation.

(1) Raising the level of environmental awareness among residents: The choice of both residents and the government to participate in waste separation is a very complex issue that depends on individual residents’ attitudes and level of environmental awareness. Agreeing to participate does not necessarily mean fully supporting household waste separation. Participation in waste separation is essential, as is the quality of individual residents’ waste separation. Many scholars have studied promoting waste separation and analyzed government educational efforts. Therefore, the government should improve the environmental awareness cultivation system by starting from both school and non-school systems, establishing a top-down waste-separation education model, and striving to create an excellent ecological awareness atmosphere in the whole society to effectively improve the level of environmental awareness of individual residents and their family members, thus achieving the goal of both increasing the willingness to participate in waste separation and enhancing the quality of individual residents’ waste separation.
(2) Strengthen government support policies and programs: As residents are sensitive to participation benefits and cost changes, participating subjects are more inclined to loss avoidance. The government should thoroughly investigate the ratio of benefits to costs of participating topics in waste classification, actively give a certain amount of reasonable financial subsidy plan to fully ensure that the benefits of participating subjects in waste classification are more significant than the costs, and at the same time optimize the waste-classification management chain, redesign the local unreasonable places, and strive to reduce the government. At the same time, we encourage the government to optimize the waste-separation management chain, redesign the local irrationalities, and try to reduce the management costs.
(3) Continuous and in-depth research on garbage sorting: In this paper, we combine the cumulative prospect theory and evolutionary game to consider the influence of the psychological changes of the participating subjects on their willingness to participate in waste separation. Moreover, due to the time limitation, current research conditions, and the number of participants, we cannot thoroughly examine the characteristics of individual residents. We intend to conduct specific research on individual residents’ factors in the subsequent studies, such as the study of residents’ waste-separation behavior based on the theory of planned behavior, the survey of residents’ waste-separation behavior under the environmental behavior model, and the study of psychological empowerment cognition on residents’ waste-separation behavior.

## Figures and Tables

**Table 1:** Game payment matrix for participation in waste-separation behavior.

	Government	Participation (y)	Non-Participation (1 − y)
Residents			
Participation (x)		U_1_ + βK − M − T U_2_ + (1 − β)K − C + P	U_1_ − M − T U_2_
Non-participation (1 − x)		U_1_ U_2_ − C	U_1_ U_2_

**Table 2:** Equilibrium results when the expected benefits for one of the residents and the government are less than the costs they pay.

Equilibrium Point	∣Je∣	trJe	Results
(0, 0)	+	-	ESS
(0, 1)	-	+	Instability point
(1, 0)	-	+	Instability point
(1, 1)	+	+	Saddle point

Note: “+” means the result of the operation is positive, “-” means the result of the operation is negative.

**Table 3:** Equilibrium results when the benefits received by residents and the government are both greater than the costs paid by them.

Equilibrium Point	∣Je∣	trJe	Results
(0, 0)	+	-	ESS
(0, 1)	+	+	Instability point
(1, 0)	+	+	Instability point
(1, 1)	+	-	ESS
$\left(\frac{C}{\left(1-\beta \right)K+P},\;\frac{M+T}{\beta K}\right)$	+	0	Saddle point

Note: “+” means the result of the operation is positive, “-” means the result of the operation is negative.

**Table 4:** Simulation parameter values.

Parameters	Meaning	Value	Parameters	Meaning	Value
x	Probability of resident participation	0.3	M	Resident opportunity cost	1
y	Probability of government involvement	0.5	T	Resident time cost	3
β	The direct benefit allocation factor	0.5	P	Potential government benefits	3
K	Direct benefits	90	C	Government management costs	6

## Data Availability

The data used to support the findings of this study will be available from the corresponding authors upon request.
